# Rhinoplasty and Functional Endoscopic Sinus Surgery

**DOI:** 10.1155/2011/473481

**Published:** 2011-07-27

**Authors:** George L. Murrell

**Affiliations:** ^1^Department of Otolaryngology-Head and Neck Surgey, Naval Hospital Camp Pendleton, CA 92055, USA; ^2^Department of Surgery, The Uniformed Services University of the Health Sciences, Bethesa, MD 20814, USA; ^3^Department of Health Care Sciences, The George Washington University School of Health Sciences, Washinton, DC 2003-7, USA

## Abstract

An increasing number of patients are opting for combining sinus surgery and cosmetic rhinoplasty. The author has been performing rhinoplasty with FESS since April of 1990. The technique and equipment used in early cases is much different than that used in more recent surgeries. Specific advances include high definition monitor, intraoperative navigation system, and powered dissecting instruments. The benefits of these advances are illustrated by a review of the more recent cases performed by the author. Combined rhinoplasty and FESS can be performed with good results (functional and cosmetic) and minimal complications. Advances in sinus surgery technique and equipment have made the procedure safer, faster, more precise, and more comfortable.

## 1. Introduction

Combining rhinoplasty and FESS was first reported in 1991 by Shemen and Matarasso [1]. Since then numerous authors have reported large studies illustrating the overall safety and efficacy of combining the two procedures [2–8]. The focus of this paper is to report the author's recent specific experience with combining rhinoplasty and FESS and highlight the evolution that has occurred in sinus surgery during the authors' 20 years of combining the procedures.

## 2. Methods

A retrospective chart review was performed on all of the author's patients who underwent combined rhinoplasty and FESS between July 2002 and October 2010. All patients underwent otolaryngologic work up which included history, head and neck exam, office rigid endoscopy, and fine cut (4 mm) CT scanning of the paranasal sinuses (axial and coronal views). All patients had been treated with oral antibiotics and nasal steroids prior to the CT scan. All patients had standard preoperative rhinoplasty photos taken.

All cases were performed as outpatient procedures by the same surgeon under general endotracheal anesthesia. All patients received IV antibiotics (cefazolin 1 gram) and IV steroids (Dexamethasone 10 mg) at the time of induction. A throat pack was positioned, and cottonoid pledgets soaked in 4 cc of 4% cocaine were placed in the nose prior to nasal injection with 1% lidocaine with 1 to 100,000 epinephrine. If septoplasty was indicated, it was performed first and nasal splints secured. Next the FESS was performed using technique adapted from Messerklinger [9], Stammberger [10], and Kennedy [11]. Equipment for the FESS included high definition monitor, 4 mm endoscopes (0 degree, 30 degrees, and rarely 70 degrees) and powered instruments/Landmarx Navigation System by Medtronic (710 Medtronic Parkway, Minneapolis, MN, 55432-5604). Cottonoid pledgets soaked in oxymetazoline were placed in the dissected sinus cavities (typically 4 on each side) at the completion of the FESS and left in place during the rhinoplasty which was performed next. Osteotomies if indicated were always performed as the last surgical maneuver. If inferior turbinate reductions were indicated, they were performed immediately prior to the osteotomies. At the conclusion of the surgery the sinus cavities were filled with two Meropacks (Medtronic) and a 4 mm Rapid Rhinos (ArthroCare, 7500 Rialto Boulevard, Building 2, Suite 100, Austin, TX 78735) was placed on each side of the nose. Patients were discharged with oral antibiotics (usually amoxicillin-clavulanic acid) and oral pain medication (usually oxycodone and acetaminophen). Patients were seen on postoperative day 1 for rapid rhino removal. Nasal sinus irrigations (at least twice a day) and a 3-day course of oxymetazoline were started in the afternoon of postoperative day 1. On postoperative day 7, septal splints, external cast and any nasal sutures were removed. Patients were then seen at 1 month, 3 months, 6 months, one year, and yearly after surgery.

## 3. Results

Between July 2002 and October of 2010, 26 patients underwent rhinoplasty combined with FESS. The specific characteristics for these patients are listed in Table 1. There were 16 females and 10 males, age range from 21–61 years, with a mean of 31.5 years. Endonasal approach was used in 22 cases and external approach used in 4 cases. The average total operative time was 110 minutes. On average, the rhinoplasty and FESS took about equal parts of this operative time (50 minutes for the FESS and 60 minutes for the rhinoplasty). The average total blood loss was 40 ccs. 

Concerning the FESS procedures, 2 patients were treated with only concha bullosa resection, 3 patients were treated with unilateral ethmoidectomy-maxillary antrostomy, 4 patients were treated with bilateral ethmoidectomy-maxillary antrostomy, 2 patients were treated with additional nasofrontal exploration, and 13 patients were treated for pan sinus disease. Therefore, most of the patients (14/26) had what would be considered extensive sinus disease, that is, involving all sinuses or maxillary and ethmoid disease with extension to the frontal and/or sphenoid sinus. All but one patient had a septoplasty. Concerning the rhinoplasty procedures, the most common aesthetic procedure was dorsal hump reduction, performed in 14 patients. It is of particular note that 14 patients had some type of cartilage grafting performed (strut, tip graft, alar batten or extension grafts, alar rim grafts, dorsal graft, nasolabial, caudal columellar graft spreader grafts, septal grafts). Auricular cartilage was harvested in 2 patients. All patients were followed for at least a year postoperatively except those operated on less than a year ago at the time of this report. All patients reported an improvement in their sinus symptoms and satisfaction with their nasal appearance. No revision rhinoplasties have been performed on this group thus far. 

**Figure 1:**
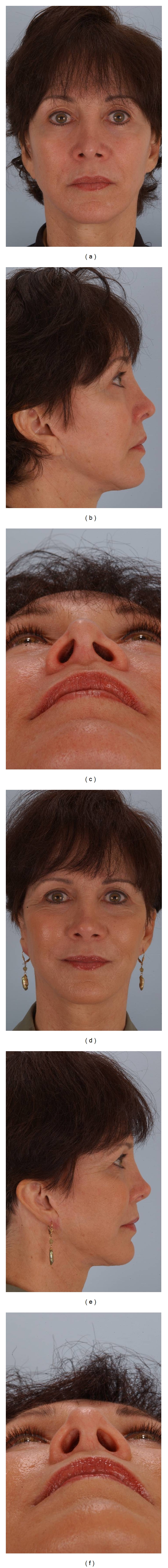
48 year old female, 15 years status post srp with alloplastic implant. Preoperative views: frontal (a) lateral (b), submental (c). One year postoperative views: frontal (d), lateral (e), submental (f).

**Figure 2:**
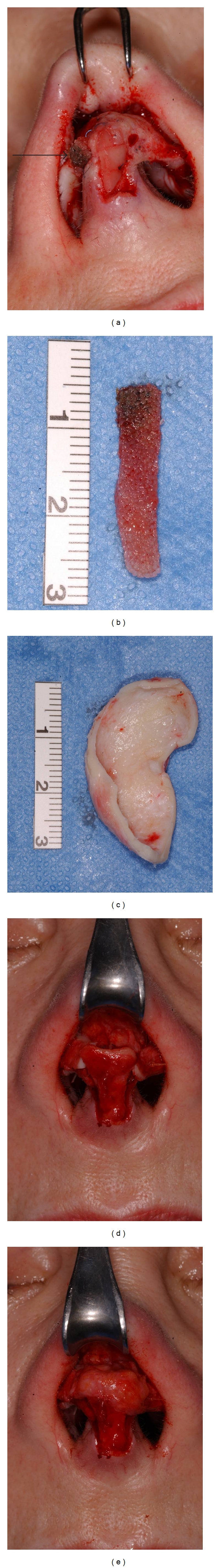
Intraoperative photographs: external approach has been performed and alloplastic implant is visible denoted by arrow (a), alloplastic implant after removal (b), conchal cartilage graft (c), alar battens and tip graft secured (d), grafts covered with conchal perichondrium (e).

There were no major complications. There were two minor complications. Patient no. 8 experienced dermatitis secondary to the adhesive used under the nasal cast. This was treated with cast removal a day early (postoperative day 6) and topical hydrocortisone cream. This patient's case is presented in Section 4 and illustrated in Figures 1 and 2. Patient no. 11 was thought to have a small intraoperative cerebrospinal fluid leak high in the left ethmoid sinus. No intraoperative diagnostic samples of the fluid were sent for laboratory analysis. The suspected area was treated with intraoperative intranasal maneuvers (mucosal transfer, gelfoam, and surgical packing) and was not present postoperatively. 

All of the cases in the 2002 to 2009 series had the FESS performed with the benefit of powered instruments and Landmark navigation system both manufactured by Medtronic. The average operative time for the FESS was 50 minutes. FESS operative times prior to powered instrument use were longer. For example, the author's average FESS operative time from October 1998 to October 1999 was 71 minutes.

## 4. Report of Cases

### 4.1. Case  1

A 48-year-old female was 15 years status post septorhinoplasty with an alloplastic implant by another surgeon (Table 1, patient no. 8). Preoperative, intraoperative, and 12-month postoperative photographs are shown in Figure 1. Intraoperative photographs are shown in Figure 2. She presented with a 2-month history of foul smell coming from the nose, yellow rhinorrhea, and implant exposure high in the right nasal vestibule. She also had chronic sinusitis with blockage of bilateral maxillary and ethmoid sinuses. She underwent FESS to clear the involved sinuses and external approach rhinoplasty with removal of the alloplastic implant. Her nasal tip was very scarred and contracted. The alloplastic implant which appeared to have been placed as an extended strut was removed. This patient required rebuilding of the nasal tip. Auricular cartilage was harvested and used to fashion a strut, alar batten grafts, and a tip graft. A perichondrial graft from the conchal cartilage was used to cover and soften the tip complex. The one-year postoperative views show a softer more natural, less pinched nasal tip, and a more favorable tip to nasal dorsum relationship. The patient's sinus symptoms and infection from the alloplastic implant all resolved after surgery.

### 4.2. Case  2

**Figure 3:**
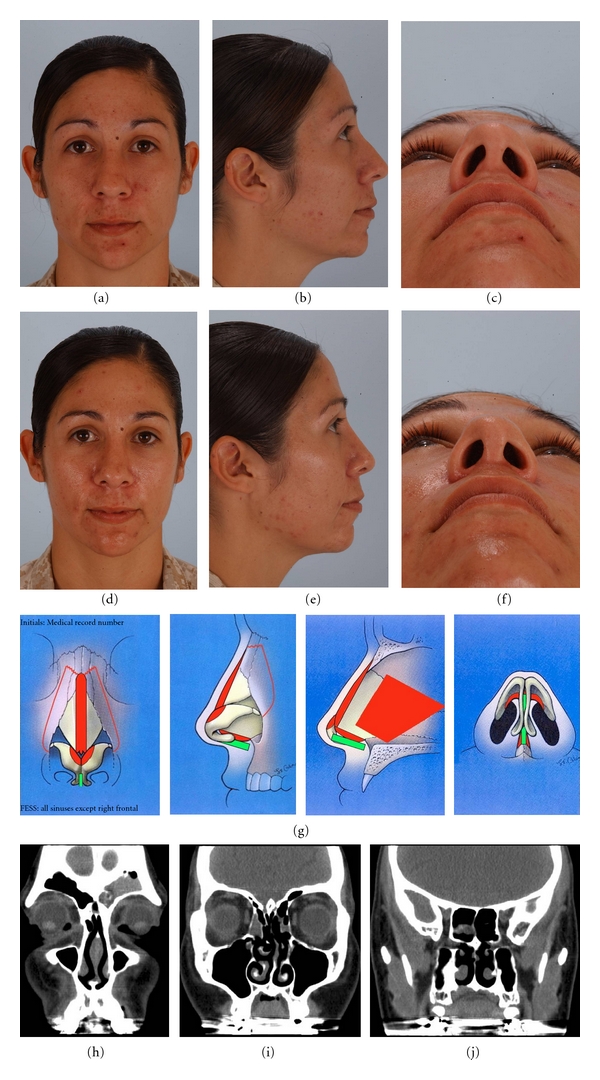
27-year-old female with chronic sinusitis who wanted a smaller more refined nose. Preoperative views: frontal (a), lateral (b), submental (c). One-year Postoperative views: frontal (d), lateral (e), submental (f). Intraoperative rhinoplasty worksheet indicating septoplasty, profile alignment, osteotomies, cephalic strip removal, and osteotomies (g). Preoperative serial coronal CT scans showing extensive sinus disease (h, i, j).

A 27-year-old female (patient no. 9, Table 1) had chronic sinusitis, nasal obstruction, with septal deviation to the left and modest cosmetic concerns. She wished to have a smaller, more refined nose. Preoperative, 12-month postoperative photographs, intraoperative rhinoplasty diagram, and preoperative sinus CT scans are shown in Figure 3. She underwent FESS to clear all the sinuses (except right frontal) and endonasal septorhinoplasty for profile alignment, modest tip refinement, and narrowing of the boney pyramid. The postoperative change in profile and nasal tip position is subtle but real. This is the finesse rhinoplasty result she desired. Her sinus symptoms resolved after surgery.

## 5. Comment

Many patients with cosmetic nasal concerns will also have functional complaints (nasal obstruction and/or sinus problems). These functional complaints should be fully evaluated. In addition, many patients with functional nasal problems would like a cosmetic nasal improvement. These desires for cosmetic improvement can and should be tactfully elicited by the nasal surgeon. It makes sense that patients who would benefit from rhinoplasty and sinus surgery would wish to combine the two procedures. Combining the procedures can save patients time, money, and inconvenience. Advances in sinus surgery have made combining rhinoplasty and FESS even more appealing.

The author has been combining rhinoplasty and FESS since 1990. Back then, to perform the FESS, one looked directly through the endoscope, no monitor. In retrospect this was a bit like performing surgery by looking though a key hole. Only a few simple instruments were used to clear the sinuses (forceps, backbiter, and suction). This required almost monotonous removal cleaning and reinsertion of instruments. It was very time consuming and also led to more blood loss. Today, a high-definition monitor offers a superior view. Powered instruments which have combined suction, irrigation, debridement, and even cautery save surgical steps, operative time and result in less blood loss. An intraoperative navigation system is a valuable tool used for anatomic confirmation. Dissolvable sinus packing (Meropacks-Medtronic) has increased patient comfort. Newer nasal packs (Rapid Rhinos-ArthroCare) cause less discomfort because they remain lubricious and do not disrupt clot formation on removal. All of these sinus surgery advances have made the procedure faster, safer, more precise, and more comfortable (for the patient and the surgeon). 

The presented series of 26 cases of rhinoplasty with FESS adds to the literature illustrating the overall safety and efficacy of combining the procedures. The metrics for the series shows that the sinus surgery portion of the case need not be overly time consuming, on average taking less than an hour. Total blood loss for the combined cases was a relatively small amount, 40 ccs. Of note is that over half (14/26) of the patients had some type of cartilage graft placed, and no infection, extrusion, malposition, or resorption occurred. 

The patient presented in Case  1 is remarkable for alloplastic graft removal. To the author's knowledge no previous cases of FESS combined with rhinoplasty involving alloplastic graft removal have been reported. 

In conclusion the author has had good results, both functional and cosmetic, combining rhinoplasty and FESS. Advances in sinus surgery have made combining the two procedures even more appealing.

##  Disclusure

The author has no financial or commercial relationships to disclose. This paper was presented at the Annual meeting of The Rhinoplasty Society, La Vegas, NV, May 2, 2009.

##  Disclaimer

The views expressed in this paper are those of the author and do not necessarily reflect the official policy of position of the Department of the Navy, Department of Defense, nor the US Government. Dr. Murrell is a military service member. This work was prepared as part of my official duties. Title 17, USC, Section  105 provides that “Copyright protection under this title is not available for any work of the United States Government.” Title 17, USC, Section  101 defines a US government work as a work prepared by a military service member of employee of the US Government as part of that person's official duties.

## Figures and Tables

**Table 1:** Characteristics of 26 patients who had combined rhinoplasty and FESS.

PT no.	Age	Sex	APP	Rhinoplasty procedure	FESS procedure	Time	EBL
1	22	F	C	S, H, O	L: E, M	76	20
2	22	M	C	S, ST	R: M	45	15
3	29	M	C	S, O	R: M, E, F	64	10
4	44	M	C	S, CS, TG, CCG, ABR, DG, ACG	B: M, E, F	143	100
5	23	M	C	S, O	B: M, E, CB	54	25
6	23	F	C	S, H, O, CS	B: M, E	88	30
7	24	M	C	S, VSR, CSR, TR	B: M, E	50	15
8	48	F	O	TG, BT, ACG, ALLX	B: M, E, SP, F	234	175
9	27	F	C	S, CS, H, O, ST	B: M, E, SP, FR, CB	165	15
10	27	F	C	S, TR, H, O, CS, CSR	B: CB	90	150
11	25	M	C	S, TR, H, O, CSR	B: M, E, SP, FR	115	150
12	40	F	C	S, H, O, CSR	R: CB	100	15
13	26	F	C	S, H, TR	L: CB	54	10
14	30	F	C	S, H, O	B: M, E, SP, FR	62	10
15	50	F	O	S, H, ST, VSR, CSR, TG, CG, ACG	B: M, R, SP, FR	277	60
16	61	F	C	S, H	B: M, E, SP, FR	58	15
17	24	F	C	S, ST, CS, CCG, NL	B: M, E, SP, FR	90	30
18	37	F	O	S, H, O, CS, TG, TR	B: M, E	168	10
19	51	F	C	VSR, CSR	B: M, E, SP, FR	61	10
20	38	F	C	H, O, CS, TG, CSR, TR	B: M, E, SP, FR	128	30
21	31	F	C	S, H, ST, CS, AR, NLP, CT, TR	B: M, E, SP, FR	208	10
22	26	F	O	S, H, ST, CS, TG, AR, ASO	B: CB	188	30
23	23	M	C	S, SG	B: M, E, SP, FR	55	5
24	23	M	C	S, SG, LCAEG, SO, TR	L: M, E	121	50
25	21	M	C	S, ST, CS, VSR, TG, CT, LCAEG	R: CB	103	10
26	24	M	C	S, SG, TR	B: M, E, SP, FR	54	31

APP: approach; C: closed/endonasal approach; O: open; C: closed; S: septoplasty; H: hump reduction; O: osteotomies; ST: strut; CS: cephalic strip resection; ABR: alar base reduction; CG: composite graft; ACG: auricular cartilage graft; VSR: vestibular skin resection; CCG: caudal columellar graft; DG: dorsal graft; TG: tip graft; CSR: caudal septal resection; TR: turbinate reduction; NLP: nasolabial plumping graft; AR: alar rim graft; CT: columellar thinning; ASO: anterior septal overlap graft; SG: spreader graft; LCAEG: lateral crural abutment extension graft, R: right; L: left; B: bilateral; E: ethmoidectomy; M: maxillary antrostomy; SP: sphenoidotomy; FR: nasofrontal exploration; CB: concha bullosa resection.
